# The Comparative Efficacy and Safety of Peginterferon Alpha-2a vs. 2b for the Treatment of Chronic HCV Infection: A Meta-Analysis

**Published:** 2010-06-01

**Authors:** Seyed Moayed Alavian, Bita Behnava, Seyed Vahid Tabatabaei

**Affiliations:** 1 Baqiyatallah Research Center for Gastroenterology and Liver Disease, Baqiyatallah University of Medical Sciences, Tehran, Iran

**Keywords:** Peginterferon Alpha-2a, Peginterferon Alpha-2b, Pegasys, PegIntron, HCV, Meta-Analysis, Systematic Review

## Abstract

**Background and Aims:**

Two types of peginterferon, alpha-2a (PEG-IFN-α2a) and 2b (PEG-IFN-α2b), are approved for the treatment of hepatitis C infection. Several high-quality studies have compared the efficacy of these two types of interferon, but it seems that any of these trials had inadequate statistical power on their own to find even a tiny difference between these two medicines. We pooled the available data in the literature to find any small difference between these two medicines.

**Methods:**

In a systematic review of the literature, randomized controlled trials comparing the use of PEG-α2a vs. 2b were assessed. The DerSimonian and Laird method was employed to run meta-analysis. The end points were virological responses.

**Results:**

In 7 randomized controlled trials, 3518 patients were randomized to receive PEG-IFN-α2a + ribavirin (n=1762) or PEG-IFN-α2b + ribavirin (n=1756). Early virological response (EVR), early treatment response (ETR), and sustained virological response (SVR) were greater for patients treated with PEG-IFN-α2a. Odds Ratios (ORs) were 1.38 (95% confidence interval [CI] 1.11-1.71), 1.67 (95% CI 1.24-2.24), and 1.38 (95% CI 1.02-1.88) respectively. In the subset of naïve patients with genotype 1/4 and 2, ORs of SVR were 1.38 (95% CI 1.02-1.88) and 4.06 (95% CI 1.67-9.86) respectively. PEG-IFN-α2a had significantly higher rate of neutropenia OR=1.50 (95% CI 1.25-1.79) but pooled OR for withdrawal rates was not significant [OR=0.78 (95% CI 0.47-1.29)].

**Conclusions:**

PEG-IFN-α2a with similar safety is more effective than PEG-IFN-α2b. A longer duration of maximum serum concentration compared with PEG-IFN-α2b (168 vs. 48-72 h.) yields a greater SVR and higher neutropenia in PEG-IFN-α2a recipients.

## Introduction

Hepatitis C virus (HCV) infection is globally a major cause of liver-related morbidity and mortality [1],[2],[3]. It is estimated that around 170-200 million individuals are living with chronic HCV infection worldwide and are at risk for hepatocellular carcinoma and cirrhosis [4], [5]. The goal of therapy is to achieve a sustained virological response (SVR),defined as an elimination of the virus that is sustained for at least 6 months after the end of treatment. Attaining SVR prevents the development of cirrhosis, liver failure and hepatocellular carcinoma (HCC), and improves the infected patients’ quality of life [6]. Interferon (IFN) alpha, an immune-response modifying agent that has a direct antiviral effect and enhances immune response to viruses, is the backbone of treatment for chronic HCV infection. However, monotherapy with IFN at 3 million units 3 times weekly for 48 weeks produces low SVR rates of 20% [7],[8],[9],[10],[11]. The addition of ribavirin, a synthetic guanosine analogue that takes direct action against RNA and DNA viruses, to the standard IFN alpha, for a 48-week regimen produces SVRs up to a suboptimal level of 40% in naïve patients [12],[13],[14]. An important recent advance in the treatment of chronic HCV was the introduction of a long-acting IFN known as peginterferon (PEG-IFN), which, in combination with ribavirin, further increases overall SVR rates up to 52% in patients with type 1, and 80% in patients with type 2 or 3 of HCV infection [15],[16],[17],[18]. PEG-IFN alpha is the product of a process called pegylation. In this process the polyethylene glycol molecule is bonded to standard IFN covalently. The polyethylene glycol (PEG) part of the compound increases the biological half-life of the IFN protein and its biological effects by slowing the rate of absorption from subcutaneous sites, and protects the IFN molecule from proteolytic breakdown. Two types of PEG-IFN alpha are available. Both are type I alpha IFN, but differ in the size and structure of the IFN and the polyethylene glycol molecules, as well as in pharmacokinetic properties. The US Food and Drug Administration has approved a fixed-dosing regimen for PEG-IFN alpha-2a (PEG-IFN-α2a) with a molecular weight of 40 kDa (180 µg once weekly) and a weight-based regimen (1.5 µg/kg once weekly) for PEG-IFN alpha-2b (PEG-IFN-α2b) with a molecular weight of 12 kDa. At present dual therapy of both PEG-IFNs and ribavirin is the standard antiviral regimen for chronic HCV infection; however, current guidelines make no recommendation for one variety of pegylated IFN (PEG- IFN) over the other, and it is unclear if there are clinically significant differences between dual therapy with PEG-IFN-α2a and with 2b. A previously published systematic review has focused on comparing dual therapy with either PEG-IFN-α2a or 2b versus dual therapy with standard IFN, and then indirectly compared these two types of PEG-IFN [19]. Since then several head-to-head randomized clinical trials (RCTs), which have directly compared dual therapy with these two types of PEG-IFN, have been published, but a system  ic review and meta-analysis of these RCTs has not been conducted yet. The purpose of this meta-analysis is to compare the advantages and disadvantages of dual therapy with PEG-IFN-α2a, with dual therapy with PEG-IFN-α2b, based on the results of head-to- head randomized controlled trials.

## Materials and Methods

### Search methods for the identification of studies

We made an electronic search of Medline, Scopus, the Cochrane Central Register of Controlled Trials, and ISI with different possible keywords for peginterferon alpha-2a and 2b. We did not apply any temporal limits. The keywords we used were different combinations of “hepatitis C virus” or “HCV” with following terms: “peginterferon alpha-2a” and “peginterferon alpha-2b”. In different queries, “pegylated interferon” replaced “peginterferon” and “alfa” replaced “alpha” to retrieve all relevant citations. In another query, the commercial brand names were used; “Pegasys” and “PegIntron”.

### Data collection and analysis

All citations were imported into an EndNote library, then titles and abstracts were screened by two separate investigators that were blind to each other’s study selection. Full texts of all selected reports were retrieved and assessed according to our predefined inclusion and exclusion criteria. Data from studies that met our criteria were extracted by two investigators separately and rechecked by a third one. The data for outcome of treatment were tabulated according to the treatment regimen (dual therapy with PEG-IFN-α2a and ribavirin or PEG-IFN-α2b and ribavirin) in excel spreadsheets. The decision to include or exclude a study, and predefined assumptions, were made and agreed to by all authors before running the meta-analysis. The data for the characteristics of the studies and patients were abstracted by standard questionnaires including first author name, journal name, methodology of randomization, allocation concealment, blindness to treatment, publication year, and sample size in each treatment arm; as well as viral loads, liver histologies and frequencies of genotypes, SVR (undetectable HCV-RNA 6 months after untreated follow-up), ETR (undetectable HCV-RNA immediately on treatment cessation), rapid virological response (RVR) (undetectable or >2Log reduction of serum HCV-RNA level after 4 weeks of therapy), EVR (undetectable or >2Log reduction HCV RNA after 12 weeks of therapy), anemia, leukopenia, thrombocytopenia, depression and severe psychiatric disorders, flue-like syndrome and treatment discontinuation according to treatment arms.

### Inclusion and exclusion criteria

Randomized controlled trials of adults with chronic HCV infection seronegative for human immunodeficiency virus (HIV) and hepatitis B virus (HBV) infection were included if study patients: 1) received PEG-IFN-α2a 180 µg per week plus ribavirin 800-1400 mg in one treatment arm and PEG-IFN-α2b 1.5 µg/kg per week plus ribavirin 800-1400 mg per day in another treatment arm, 2) were treated for at least 24 weeks if infected with HCV genotypes 2 or 3, and for at least 48 weeks if infected with genotypes 1 or 4 and The diagnosis of chronic HCV infection required a detectable HCV RNA value and a duration of at least 6 months of infection. Articles in all languages that met the criteria were included. Inclusion of patients with previous history of treatment, study dose modification, administration of growth factors, and antidepressants was allowed. Studies were excluded if study patients: 1) had decompensated liver disease, 2) had positive seromarkers for HIV or HBV infection 3) were not all accounted for at the end of the study, 4) had significant co-morbidities, and 5) received lower than 1.5 µg/kg PEG-IFN-α2b or 180 µg PEG-IFN-α2a. Quasi-experimental trials and observational studies were excluded as well.

### End points of interest

The primary end point for comparison of efficacy was SVR, defined as undetectable HCV-RNA for the 6 months after treatment cessation. The secondary end points of interest were: RVR defined as undetectable, or a reduction of more than 2log10 HCV-RNA after 4 weeks of treatment; early virological response (EVR) defined as undetectable, or a reduction of more than 2log10 HCV RNA at week 12 of treatment; and end of treatment response (ETR) defined as undetectable HCV RNA at the end of the course of treatment. The primary end points for comparison of safety were withdrawals and dropouts. The secondary end points were dose modifications, adverse events including flu-like syndrome and laboratory abnormalities defined as Hb < 10 g/dL, neutropenia (< 750 c/mm3) and thrombocytopenia (<50,000 c/mm3).

### Assessment of methodological quality

Methodological quality, defined as confidence that the design and report will limit the chance of bias in intervention comparison, was evaluated, as previously reported [20]. Allocation sequence generation, allocation concealment and blinding were extracted as measures of bias control. The allocation sequence generation was considered adequate if based on a table of random numbers or on computer-generated random numbers. The allocation concealment was considered adequate if patients were randomized through a central independent unit or using serially numbered opaque sealed envelopes or something similar. Blinding was described as adequate if the trial was described as double-blind, and both patients and investigators were unaware of the allocated treatment. To assess the risk of bias further, we also extracted the number and reasons for dropouts and withdrawals. Conflicts were resolved by consensus.

### Source of support

This meta-analysis was not supported by any pharmaceutical company or government agency, or grants from other sources.

### Data synthesis

All analyses were performed in Stata 10, (Stata Corp. College Station, TX, USA). Data on all randomized patients were included, based on the intention-to-treat principle, irrespective of compliance or follow-up. To manage missing data, we used worst-case scenario analysis and, since we had a positive outcome (virological response), all missing data were counted as non-responders. Subgroup analyses on the SVR of naïve patients with genotype 1 or 4 and patients with genotypes 2 and 3 were performed. The results are presented as an Odds ratio (OR) with a 95% confidence interval. Meta-analysis was performed, using the random effects model of the DerSimonian and Laird method. The random effects model provides a more conservative estimate of significance. This model operates under the assumption that included studies are only a random sample of all studies that will be conducted, so that heterogeneity among individual studies will result in a wider CI of the summary estimate. Therefore, using the DerSimonian and Laird random effects model, the reported summary estimate was calculated as an average of the individual study results weighted by the inverse of their variance [21]. The estimate of heterogeneity was taken from the Mantel-Haenszel model; under the null hypothesis of the test of heterogeneity, there is no difference in treatment effect among groups (this follows a N(2) distribution with k-1 degree of freedom, where k is the number of studies contributing to the meta-analysis). Study results were considered heterogeneous if the resultant P-value was less than 0.1 [22]. I2 was also used to provide a measure of the degree of inconsistency among the studies’ results. Its quantity describes the percentage of total variation across studies that is due to heterogeneity rather than chance. I2 lies between 0% and 100%. A value of 0% indicates no observed heterogeneity, and larger values show increasing heterogeneity [23].

## Results

### Results of the search

**Figure 1:**
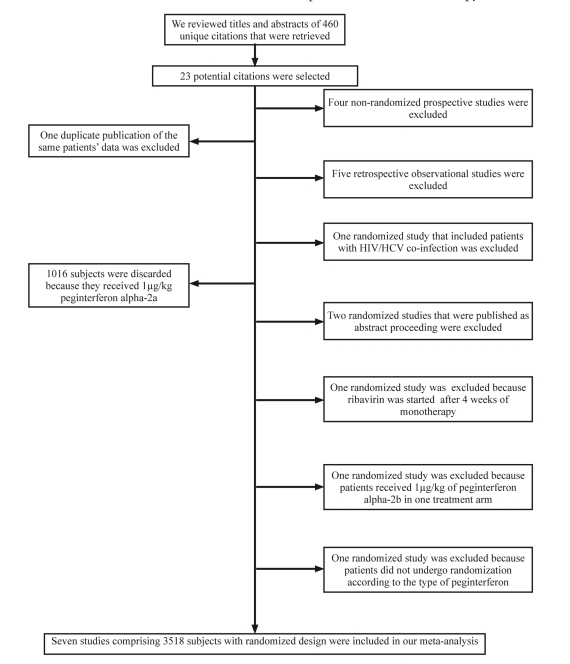
Analysis of search results

Our search strategy yielded 460 unique citations that included fourteen randomized clinical trials [24][25][26][27][28][29][30][31][32][33][34][35][36][37] four prospective [38][39][40][41] and five retrospective studies [42][43][44][45][46]that compared PEG-IFN-α2a plus ribavirin with PEG-IFN-a2b plus ribavirin (Fig 1). Among fourteen studies with randomized design, two studies were excluded because they were published as abstract proceedings [35][36],one study was excluded because patients received 1 µg/kg of PEG-IFN-a2b [31],another study was excluded because patients received ribavirin after 4 weeks of monotherapy with PEG-IFN-α2a or 2b [30], another was excluded because patients did not undergo randomization according to type of PEG-IFN, but according to standard treatment duration of either PEG-IFNs against individualized treatment duration based on viral kinetics [34], another one was excluded because it included patients with HIV/HCV co-infection [32]. One thousand-sixteen subjects in one treatment arm of the study by McHutchison et al. were also excluded because patients received 1 µg/kg of PEG-IFN-α2b. One duplicate publication of the same patients’ data was also excluded [25]. At the end, 7 randomized clinical trials were included in a meta-analysis [24],[29],[33],[37] Fig 1. In two studies by Sporea and Di Bisceglie et al. only data of RVR or EVR were available for analysis (247 subjects in PEG-IFN-α2a arm and 249 in PEG-IFN-α2b arm).

### Included studies

Study characteristics are presented in table 1 of seven included studies, three were from Italy, two from the USA and one each were from Turkey and Romania. All studies were published as full text in peer-reviewed journals between 2006 and 2010. One study by Scotto et al. included only non-responders to previous combination therapy of standard IFN and ribavirin. Sporea et al. included both naive patients and relapsers or non-responders to standard IFN and ribavirin. Patients in other trials were naïve and did not have a history of anti-HCV therapy. All included trials had randomized design. Three studies by Di Bisceglie, Sporea and Yenice et al. did not declare the method of randomization but randomization was adequate in other studies (computer generated random numbers, in studies by Ascion and Rumi et al., a table of random numbers in the study by Scotto et al. and an interactive voice system in the study by McHutchison). Only the study by McHutchison was double-blind and a multi-center trial and the other trials were single-center and open-labeled. Allocation concealment was unclear in studies by Yenice and in Rumi et al.’s study. All trials had similar inclusion criteria. The diagnosis of chronic hepatitis C was based on the presence of hepatitis C virus RNA in the blood, elevated plasma transaminases for at least six months, and a pre-treatment liver biopsy showing evidence of chronic viral hepatitis. Exclusion criteria were also very similar in all the trials and consisted of decompensated liver disease, autoimmune hepatitis, chronic hepatitis B, significant co-morbidities such as infections, kidney disease, cardiovascular disease, psychiatric illnesses, poorly-controlled diabetes, seizures, or hematological diseases with anemia, low platelets, or neutropenia as well as ongoing alcohol or drug abuse, or pregnancy. Patients with HIV co-infection were also excluded.

**Table1:** Study characteristics

**Reference No.**	**Author**	**Publication Year**	**Countries of Samples' Origin**	**Randomization**	**Allocation Concealment**	**Blinding**	**Naïve**
[24]	Ascione *et al.*	2010	Italy	Ad	Ad^a^	No	Yes
[26]	Scotto *et al.*	2008	Italy	Ad	Ad	No	No
[27]	McHutchison *et al*.	2009	USA	Ad	Ad	Ad	Yes
[28]	Rumi *et al.*	2010	Italy	Ad	Ad	No	Yes
[29]	Yenice *et al.*	2006	Turkey	NR	NR^a^	No	Yes
[31]	Di Bisceglie *et al.*	2007	USA	NR	Ad	No	Yes
[33]	Sporea *et al*.	2006	Romania	NR	NR	No	Yes/No

^a^ Ad: adequate; NR: not reported

### Patient characteristics

Three thousand five hundred and eighteen patients were randomized to receive 180 µg PEG-IFN-α2a subcutaneously per week and 800-1400 mg of ribavirin per day (n=1762) or 1.5 µg/kg PEG-IFN-α2b subcutaneously per week plus 800-1400 mg of ribavirin per day (n=1756). Patients’ characteristics are presented in table 2. Baseline patients’ characteristics were similar between cohorts in both treatment armsTable 2.Among patients who received PEG-IFN-α2a, the mean age in the subject cohort ranged from 45 to 52 years of age; gender distribution ranged from 27 to 61% male; hard-to-treat HCV types of 1/4 ranged from 52 to 100%; viral load ranged from 570 ×103 to 3.1 × 106;, and the proportion of patients with cirrhosis ranged from 18 to 20%. In PEG-IFN-α2b recipients, the mean age ranged from 45 to 53 years of age; gender distribution ranged from 27 to 60% male; hard-to-treat HCV types of 1/4 ranged from 52 to 100%; viral load ranged from 604 × 103 to 3.1 × 106 and rate of cirrhosis ranged from 16 to 18%. All those studied included only naïve patients that did not have a history of previous anti-HCV treatment, except for two studies by Scotto and Sporea et al. that included 193 subjects who were non-responders or relapsers to a previous combination therapy of standard IFN and ribavirin (81 patients were retreated with PEG-IFN-α2a).

**Table 2:** Patients characteristics

**Characteristics**	**Ascione *et al*. [24]**		**Scotto *et al*. [26]**		**McHutchison *et al*. [27]**		**Rumi *et al*. [28]**		**Yenice *et al*. [29]**		Di Bisceglie *et al*.[31]		Sporea *et al*.[33]	
	**2a**	**2b**	**2a**	**2b**	**2a**	**2b**	**2a**	**2b**	**2a**	**2b**	2a	2b	2a	2b
Patients (n)	160	160	71	72	1019	1035	212	219	37	37	189	191	58	58
Mean age	51	49	46 ± 9	48 ± 10	48 ± 8	48 ± 8	52 ± 12	53 ± 12	50	51	47 ± 1	48 ± 1	NR^b^	NR
Male (%)	51%	59%	59%	56%	59%	60%	60%	55%	35%	27%	64%	71%	NR	NR
Genotype 1/4	66%	66%	80%	73%	100%	100%	52%	52%	100%	100%	100%	100%	NR	NR
Mean HCV-RNA level (IU/mL)	570 ×10^3^	604 ×10^3^	2.4 ± 5 × 10^6^	2.1 ± 3 × 10^6^	1.9 × 10^6^	1.9 × 10^6^	2.6 ± 5.8 × 10^6^	2.2 ± 4.7 × 10^6^	NR	NR	3.1× 106	3.1× 106	NR	NR
Mean ALT (IU/mL)^a^	NR	NR	187 ± 25	175 ± 18	81%	81%	130 ± 105	129 ± 104	70%	76%	53 ± 3	65 ± 4	NR	NR
Cirrhosis (%)	21%	16%	18%	18%	NR	NR	20%	18%	NR	NR	15%	15%	NR	NR

^a^ NR:not reported.

^b^ Percentage shows proportion of patients with elevated liver enzymes

### A comparison of the efficacy of PEG-IFN-α2a and PEG-IFN-α2b dual therapy with ribavirin in HCV- infected patients

**Figure 2:**
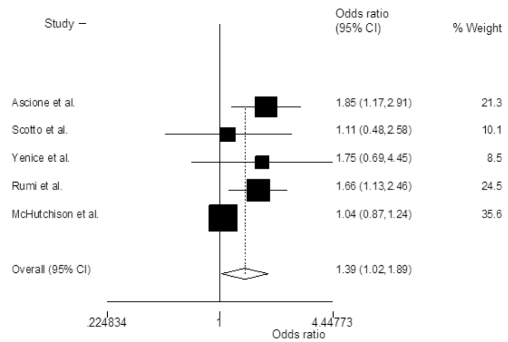
Summary estimate of Odds ratios of achieving sustained virological response (SVR) with 95% CI in patients who were treated with peginterferon alpha-2a plus ribavirin versus those treated with peginterferon alpha-2b plus ribavirin

**Figure 3:**
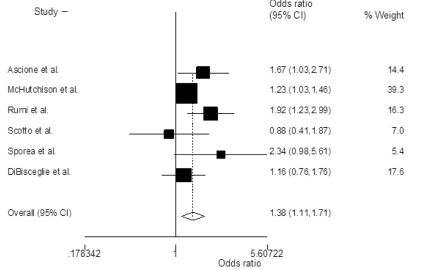
Summary estimate of Odds ratios of achieving early virological response (EVR) with 95% CI in patients who were treated with peginterferon alpha-2a plus ribavirin versus those treated with peginterferon alpha-2b plus ribavirin

**Figure 4:**
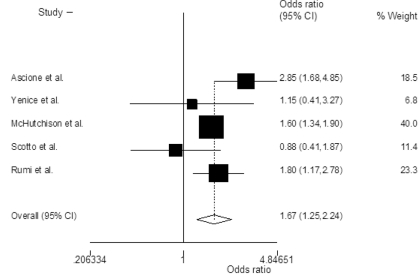
Summary estimate of Odds ratios of achieving end of treatment virological response (ETR) with 95% CI in patients who were treated with peginterferon alpha-2a plus ribavirin versus those treated with peginterferon alpha-2b plus ribavirin

The probability of achieving SVR was higher in patients treated with PEG-IFN-α2a and ribavirin when compared with PEG-IFN-α2a and ribavirin, with an OR of 1.38 (95% CI 1.02-1.88; P=0.03)(Fig 2). Heterogeneity was significant among the included studies (P=0.05, I2=55%). The odds of achieving early(Fig 3)and end of treatment virological response(Fig 4)were also higher with PEG-IFN-α2a and ribavirin, with OR of 1.38 (95% CI 1.11-1.71; P=0.003, I2=29%) and 1.67 (95% CI 1.25-2.24; P=0.001, I2=47%) respectively. There was no discrepancy between rapid virological response rates [OR=1.00 (95% CI 0.77-1.30), chi2(2)=3.4, I2=41%]. In the subset of naïve patients with genotype 1/4 and 2 infection, OR of achieving SVR was also higher in those patients who received PEG-IFN-α2a plus ribavirin(Table 3).

**Table 3:** OR of achieving SVR in subset of naïve patients who received peginterferon alpha-2a against peginterferon alpha-2b

**HCVType**	**No. Patients**	**OR(95% CI)**	**Heterogeneity Assessment**			
			***X^2^(df)***	***Τ^2^***	***I^2^***	***P***
Genotype 1/4	2715	1.36 (1.01-1.88)	7.6 [5]	0.04	34%	0.1
Genotype 2	242	4.06 (1.67-9.86)	0.09 [1]	0.00	0	0.7
Genotype 3	102	1.04 (0.47-2.32)	0.2 [3]	0.00	0	0.9

### A comparison of safety of PEG-IFN-α2a and PEG-IFN-α2b dual therapy with ribavirin in HCV- infected patients

**Figure 5:**
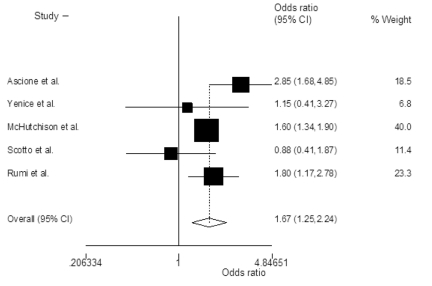
Summary estimate of Odds ratios of discontinuing the treatment with 95% CI in patients who were treated with peginterferon alpha-2a plus ribavirin versus those treated with peginterferon alpha-2b plus ribavirin

Withdrawal:All studies had sufficient information to enable comparison of the treatment discontinuation rates of those patients being treated with PEG-IFN-α2a plus ribavirin, and those treated with PEG-IFN-α2b plus ribavirin. Only patients who discontinued treatment because of severe adverse events or laboratory abnormalities were considered withdrawal data. Patients with an insufficient viral response, or those who did not return for other reasons were considered non-responders, and not included in the patient withdrawal data. Patients with both treatment regimens had similar likelihood of treatment discontinuation caused by laboratory abnormalities or severe clinical adverse events. The OR was 0.75 (95% CI 0.42-1.34) (Fig 5). The heterogeneity was significant (P=0.02, I2=64%). Further analyses were completed to examine patient withdrawals because of adverse effects and abnormal laboratory tests. The difference in withdrawal rates due to adverse events and laboratory abnormalities was not significant, with ORs of 0.72 [(95% CI 0.35-1.47), I2=63%] and 0.42 [(95% CI 0.06-2.71), I2=50%] respectively. Dose Modifications: Adequate data about dose modifications were available in all studies except Yanice et al.’s study. The discrepancy between dose modification rates of PEG-IFN or ribavirin was not significant between the two treatment regimens [OR=0.99(95% CI 0.85-1.15). No heterogeneity was observed among the studies (P=0.6, I2=0). The difference between dose modification of these two types of PEG-IFN due to side effects or laboratory abnormalities was also non-significant with low heterogeneity [OR= 1.17 (95% CI 0.94-1.46), I2=0%] Side effects and laboratory abnormalities: Sufficient information was available to enable comparison of the anemia, thrombocytopenia, neutropenia, depression or severe psychiatric complications and flu-like syndrome rates between the patients treated with PEG-IFN-α2a plus ribavirin, and those treated with PEG-IFN-α2b plus ribavirin. The ORs were 0.98 (95% CI 0.84-1.15, I2=0%) for anemia;  .37 (95% CI 0.73-2.58, I2=0%) for thrombocytopenia; 0.88 (95% CI 0.67-1.15, I2=0%) for depression or severe psychiatric complications; and 0.61 (95% CI 0.36-1.02, I2=85%) for flu-like syndrome in those patients who were treated with PEG-IFN-α2a compared with those treated with PEG-IFN-α2b. PEG-IFN-a2a had a higher rate of neutropenia [OR=1.50 (95% CI 1.25-1.79, I2=0%)].

## Discussion

Pegylation is the process of covalent attachment of polyethylene glycol polymer chains to another molecule, normally a drug or therapeutic protein. PEG-IFN is the most available commercial product of the pegylation process. The antiviral and immunomodulatory activity of PEG-IFN and the unmodified form of IFN are similar in terms of antiviral activity and receptor binding but are augmented in pegylated form. The covalent attachment of polyethylene glycol to IFN alpha protein, by increasing the molecular weight of IFN, has provided several significant pharmacological advantages over the unmodified form of IFN, such as: reduced dosage frequency without diminished efficacy with potentially reduced toxicity, extended circulating life by reducing renal clearance and enhanced protection from proteolytic degradation and increased IFN molecule stability [47][48]. The structure and size of the polyethylene glycol moiety and the means of covalent attachment play an important role in defining the properties of the modified IFN alpha [49]. PEG-IFN-α2b is obtained by the covalent linking of a linear 12 kDa PEG chain to IFN-a2b. In contrast PEG-IFN-α2a has a 40 kDa polyethylene glycol moiety, comprising two 20kDa chains [50]. These differences in the polyethylene glycol moiety and the position of pegylation results in significant differences in the pharmacodynamic and pharmacokinetic properties of the two drugs that can underlie their differences in viral dynamics and antiviral activity. PEG-IFN-α2b is a pro-drug that releases IFN alpha-2b, which behaves in the same way as standard IFN alpha in terms of its receptor binding, antiviral activity and pharmacokinetic properties [51]. In contrast, the entire pegylated molecule of PEG-IFN-α2a circulates intact and interacts with the cell surface receptors [52].

PEG-IFN-α2a is absorbed more slowly than PEG-IFN-α2b; therefore maximum concentrations occur later than with PEG-IFN-α2b, but because its molecules circulate intact, and the maximum concentration sustains up to 168 hours vs. 48-72 hours for PEG-IFN-α2b. Therefore some authors have suggested twice-weekly administration of PEG-IFN-α2b in some patients[31][53]. Since 2006, some RCTs have compared the antiviral activity of PEG-IFN-α2a and 2b in clinical settings in terms of virological responses at weeks 4, 12, 48 and 72 after the beginning of therapy, and the safety profile, including the rate of treatment withdrawals and dose modifications as a result of adverse events or hematologic abnormalities. By the aggregation of these trials, we found a similar pattern of superiority of PEG-IFN-α2a over 2b in term of SVR, ETR,EVR but not RVR. The differences in probabilities were 6% (95% CI 1-12%) for SVR, 10% (95% CI 4-15) for ETR and 7% (95% CI 3-10) for EVR, in favor of PEG-IFN-α2a. The pooled difference for likelihood of RVR was 0.00 (95% CI -5 to 5) comprising data from 2865 patients. The likelihood of SVR was also greater in PEG-IFN-α2a vs. 2b in the subset of naïve patients with both hard-to-treat HCV types: genotype 1/4 [6% (95% CI 0-  )] and genotype 2 [14% (95% CI 6-22)]. Bruno et al., in a randomized controlled trial, compared patients’ hepatitis C viral dynamics during the first 12 weeks of therapy with PEG-IFN-α2a or 2b, and revealed the same result as ours. The difference in HCV-RNA levels was not significant at week 4 of treatment, but was significantly lower at week 12 of treatment in patients who received PEG-IFN-α2a [31]. It is a very important point that the trend was toward PEG-IFN-α2a, although in the majority of single-study results, it did not reach statistical significance. It is important to note that those two studies that showed the significant advantage of PEG-IFN-α2a over 2b were published less than 2 months ago, so every narrative review or meta-analysis that has been done thus far, has concluded that there is a similarity in antiviral activity in both PEG-IFNs [19][54]. Our aggregation of the data for a safety profile of PEG-IFN-α2a and 2b showed that discrepancies in dose modification and treatment withdrawal in both types of PEG-IFN were not significant in total, but neutropenia < 750 c/mm3 was 1.5 times higher in PEG-IFN-α2a, with no observed heterogeneity among studies. This finding is confirmed in a study by Antonini et al. [55]. Lower clearance of PEG-IFN-α2a and longer duration of its maximum serum concentration could justify this finding [31].

Our meta-analysis has some significant advantages. Firstly, all of the included studies had a randomized design, and as presented in Table 2, patients in both treatment arms in all studies were remarkably homogeneous, so the results of any single study could not simply be attributed to selection bias and differences in patients’ baseline characteristics. Secondly, in addition to within-study homogeneity, there was significant homogeneity regarding laboratory abnormalities, common side effects and dose modifications. The observed heterogeneity for comparative treatment discontinuation and flu-like syndrome could be due to different patients’ ethnicity as well as to discordant host and environmental factors.

The modest methodological quality of the included studies is the only limitation of the current meta-analysis. Only the study by McHuchison was double blind and allocation concealment was unclear in studies by Yenice and Sporea et al. and random sequence generation was not declared in three studies (Table 1). Methodological research has shown that without adequate allocation concealment and blindness, even properly developed random allocation sequences can be subverted [56]. Significant inter-study homogeneities and the nature of the final outcome (surrogate) make it less possible that the pooled comparative estimate of any virological responses was influenced by lack of blindness of patients and investigators. Furthermore, the trials which adequately reported methodological quality items are large, and dominate the pooled estimates of effect. Therefore, it is unlikely that pooled estimates are biased. Another limitation of this work was lack of, or insufficiency of, data available in the literature regarding genotypes other than genotype 1/4 and relapsers or non-responders to IFN monotherapy or to therapy in combination with ribavirin.

## Conclusions

PEG-IFN-α2a, with similar safety, is more effective than PEG-IFN-α2b. A longer duration of maximum serum concentration compared with PEG-IFN-α2b (168 vs. 48-72 h.) yields greater SVR and higher neutropenia in PEG-IFN-α2a recipients.
